# Supplementary material for: The Systems Biology Research Tool: evolvable open-source software
Source: BMC Syst Biol. 2008 Jun 29;2:55. doi: 10.1186/1752-0509-2-55 (PMC2446383; doi:10.1186/1752-0509-2-55)
Supplement: Additional file 1 — SBRT Archive. An archive of the current version of the Systems Biology Research Tool. [file 1752-0509-2-55-S1.zip › sbrt-1.4.0/doc/developers_guide/api/sbrt/kernel/math/stat/comp/TauResult.html]

TauResult


|  |  |  |
| --- | --- | --- |
| **Overview** \| **Package** \| **Class** \| **Use** \| **Tree** \| **Deprecated** \| **Index** \| **Help** | |  |
| **PREV CLASS**   NEXT CLASS | **FRAMES**    **NO FRAMES**     **All Classes** |  |
| SUMMARY: NESTED \| FIELD \| CONSTR \| METHOD | DETAIL: FIELD \| CONSTR \| METHOD |  |


---


## sbrt.kernel.math.stat.comp Class TauResult

```
java.lang.Object
  sbrt.kernel.math.stat.comp.TauResult
```

**All Implemented Interfaces:**: CorrResult

---

``` public class TauResult extends java.lang.Object implements CorrResult ```

This class is used to store the results of a correlation computation.

**Author:**
:   This class was written and documented by Jeremiah Wright while in the
    Wagner lab.

---

**Constructor Summary**

`TauResult(double statistic,
double zApproximation,
int n)`
  
          Constructs a new correlation results.


| **Method Summary** | |
| --- | --- |
| `boolean` | `equals(java.lang.Object obj)`             Indicates if the provided object is equal to this correlation results object. |
| `double` | `getApproxZ()`             Returns the Z value approximated from the computed correlation statistic. |
| `double` | `getCoeff()`             Returns the computed correlation statistic. |
| `int` | `getN()`             Returns the sample size. |
| `java.lang.String` | `toString()`             Returns a string represenation of these correlation results. |

**Methods inherited from class java.lang.Object**

`clone, finalize, getClass, hashCode, notify, notifyAll, wait, wait, wait`

**Constructor Detail**

### TauResult

```
public TauResult(double statistic,
                 double zApproximation,
                 int n)
```

:   Constructs a new correlation results.

    **Parameters:**: `statistic` - the computed correlation statistic.: `zApproximation` - the Z value approximated from the computed correlation statistic.: `n` - the size of the sample.


**Method Detail**

### getCoeff

```
public double getCoeff()
```

:   Returns the computed correlation statistic.

    :   **Specified by:**: `getCoeff` in interface `CorrResult`
    :   **Returns:**: the computed correlation statistic.

---


### getApproxZ

```
public double getApproxZ()
```

:   Returns the Z value approximated from the computed correlation statistic.

    :   **Returns:**: the Z value approximated from the computed correlation statistic.

---


### getN

```
public int getN()
```

:   Returns the sample size.

    :   **Returns:**: the sample size.

---


### toString

```
public java.lang.String toString()
```

:   Returns a string represenation of these correlation results.

    :   **Overrides:**: `toString` in class `java.lang.Object`
    :   **Returns:**: a string represenation of these correlation results.

---


### equals

```
public boolean equals(java.lang.Object obj)
```

:   Indicates if the provided object is equal to this correlation results
    object.

    :   **Overrides:**: `equals` in class `java.lang.Object`
    :   **Parameters:**: `obj` - the object with which to compare. **Returns:**: `true` if `obj` is an instance of `CorrelationResults` and its statistic, Z value, and sample size are the same as this object's; `false` otherwise.


---


|  |  |  |
| --- | --- | --- |
| **Overview** \| **Package** \| **Class** \| **Use** \| **Tree** \| **Deprecated** \| **Index** \| **Help** | |  |
| **PREV CLASS**   NEXT CLASS | **FRAMES**    **NO FRAMES**     **All Classes** |  |
| SUMMARY: NESTED \| FIELD \| CONSTR \| METHOD | DETAIL: FIELD \| CONSTR \| METHOD |  |


---
